# *Lhx9* specifically modulates phalanx number, whereas *Lhx2* constrains digit length in tetrapods

**DOI:** 10.1073/pnas.2605261123

**Published:** 2026-10-20

**Authors:** Yining Tang, Shibin Bai, Shanshan Li, Ying Mao, Wanyi Dang, Huiling Xue, Songyang Shang, Ziqiu Jia, Xiaoping Li, Yanlei Liu, Xiaona Xu, David M. Irwin, Junpeng Zhang, Shuyi Zhang, Zhe Wang

**Affiliations:** ^a^ College of Animal Science and Veterinary Medicine, Shenyang Agricultural University, Shenyang 110866, China; ^b^ School of Basic Medical Science, Chengde Medical University, Chengde 067000, Hebei, China; ^c^ Department of Laboratory Medicine and Pathobiology, University of Toronto, Toronto, ON M5S 1A8, Canada

**Keywords:** tetrapod, LIM homeobox gene, expression evolution, functional divergence, digit development

## Abstract

Most nonmammalian tetrapods exhibit more than three phalanges in their posterior digits (III–V), whereas mammals are restricted to no more than three. So far, the molecular mechanism underlying this evolutionary reduction and the functions of *Lhx2* and *Lhx9* in digit development remain unclear. Here we show divergent evolution of *Lhx2* and *Lhx9* expression patterns in embryonic limbs across tetrapods. We further demonstrate that although ectopic overexpression of each gene in chick embryos delayed limb skeletal development, only *Lhx9* reduced the phalanx number in the posterior digits. Transcriptomic analysis showed that *Lhx2* regulates pathways for chondrocyte and osteoblast differentiation, and *Lhx9* preferentially influences skeletal segmentation via the bone morphogenetic protein (BMP) pathway. The unique function and differential expression of *Lhx9* in the limb buds between mammals and nonmammalian tetrapods suggest it may have acquired a role in suppressing phalanx number, driving the evolutionary reduction of phalanx number in mammals.

## Significance

Our study provides a molecular explanation for the transition in tetrapod digit evolution from the nonmammalian condition (typically more than three phalanges in the posterior digits) to the mammalian condition (no more than three). We found that *Lhx9*, rather than *Lhx2*, decreases chicken phalanx number from four or five to the mammalian two or three, with a mammalian-specific expression pattern in the posterior region of limb buds. Transcriptomic and validation experiments further elucidated the shared and unique molecular mechanisms of these paralogs. Our work connects gene expression evolution with functional validation and macroevolutionary innovation, proposing how duplicated gene divergence drives morphological specialization in tetrapods.

Tetrapods typically show more than three phalanges in their posterior digits, a condition widespread in nonmammalian vertebrates but it is generally reduced to two or three phalanges in mammals (1–3). During digit development, successive phalanges are generated in a proximal-to-distal sequence originating from the phalanx-forming region (PFR), a distal domain that provides progenitor cells at the tip of the growing digit (4, 5). Although experiments have identified several genes regulating phalanx number—such as those involved in fibroblast growth factor (FGF) signaling, *Tbx2/3*, and *Twist2*—these genes display no substantial expression differences between mammalian and nonmammalian species (6–9), with the exception of dolphins, where prolonged FGF signaling has been associated with hyperphalangy (10–12). Consequently, the molecular mechanism underlying the specific evolutionary reduction of phalanges in mammals remains unknown.

*Lhx2* and *Lhx9* are LIM-homeobox transcription factors expressed in developing limbs and are recognized as key regulators with functional redundancy (13, 14). Functional deficiency studies confirmed their critical roles: in chicks, a dominant-negative *Lhx2* caused failure of apical ectodermal ridge (AER) formation and consequent limb truncation (15), while in mice, individual knockouts of *Lhx2* or *Lhx9* did not yield morphological changes, whereas the double knockout of these two genes resulted in embryos that died around 15.5 dpc with severe defects in the entire limbs (14). Consistently, a heterozygous missense variant in *Lhx9* in humans results in finger and toe abnormalities (16). However, distinct evolutionary roles are suggested by their differential expression patterns in fish and avian lineages (17–20)—contrasting with their overlapping expression in mice (14)—and their divergent functions in gonadal development (21). Thus, despite their known collective importance, the specific, independent contributions of *Lhx2* and *Lhx9* to limb development remain to be elucidated.

To examine the independent roles of *Lhx2* and *Lhx9* in limb development, we first characterized the evolution of the gene expression patterns of these two genes. Second, we demonstrate a unique function for *Lhx9* in reducing the phalanx number, as well as its overlapping functions with *Lhx2*. Last, we analyzed and compared transcriptomic changes after ectopic overexpression of *Lhx2* and *Lhx9*. Our results identify innovative functions that *Lhx2* and *Lhx9* acquired after their origin by gene duplication, address a knowledge gap in the independent roles of *Lhx2* and *Lhx9* in limb development, and provide a molecular basis for the diversity of digit patterns in tetrapods.

## Results

### Phalangeal Patterns in Mammalian vs. Nonmammalian Tetrapods.

To investigate the evolution of phalanx number in tetrapods, we reconstructed the ancestral states for phalangeal patterns of the fore- and hindlimbs across three major clades: mammals, sauropsids, and amphibians. Results from maximum likelihood (ML) analyses revealed two key evolutionary transitions in the posterior digits: 1) In the forelimbs, the ancestral and some extant sauropsids have more than three phalanges, whereas ancestral and extant mammals and amphibians typically exhibit zero to three phalanges (*SI Appendix*, Fig. S1*A*); 2) In the hindlimbs, most ancestral and extant nonmammalian tetrapods have more than three phalanges, while ancestral and extant mammals typically possess only three (*SI Appendix*, Fig. S1*B*). This ancestral state reconstruction confirms that phalangeal patterns diverge markedly between mammalian and nonmammalian tetrapods.

### Divergent Expression of *Lhx2* and *Lhx9* in Tetrapod Limb Buds.

**Fig. 1.**
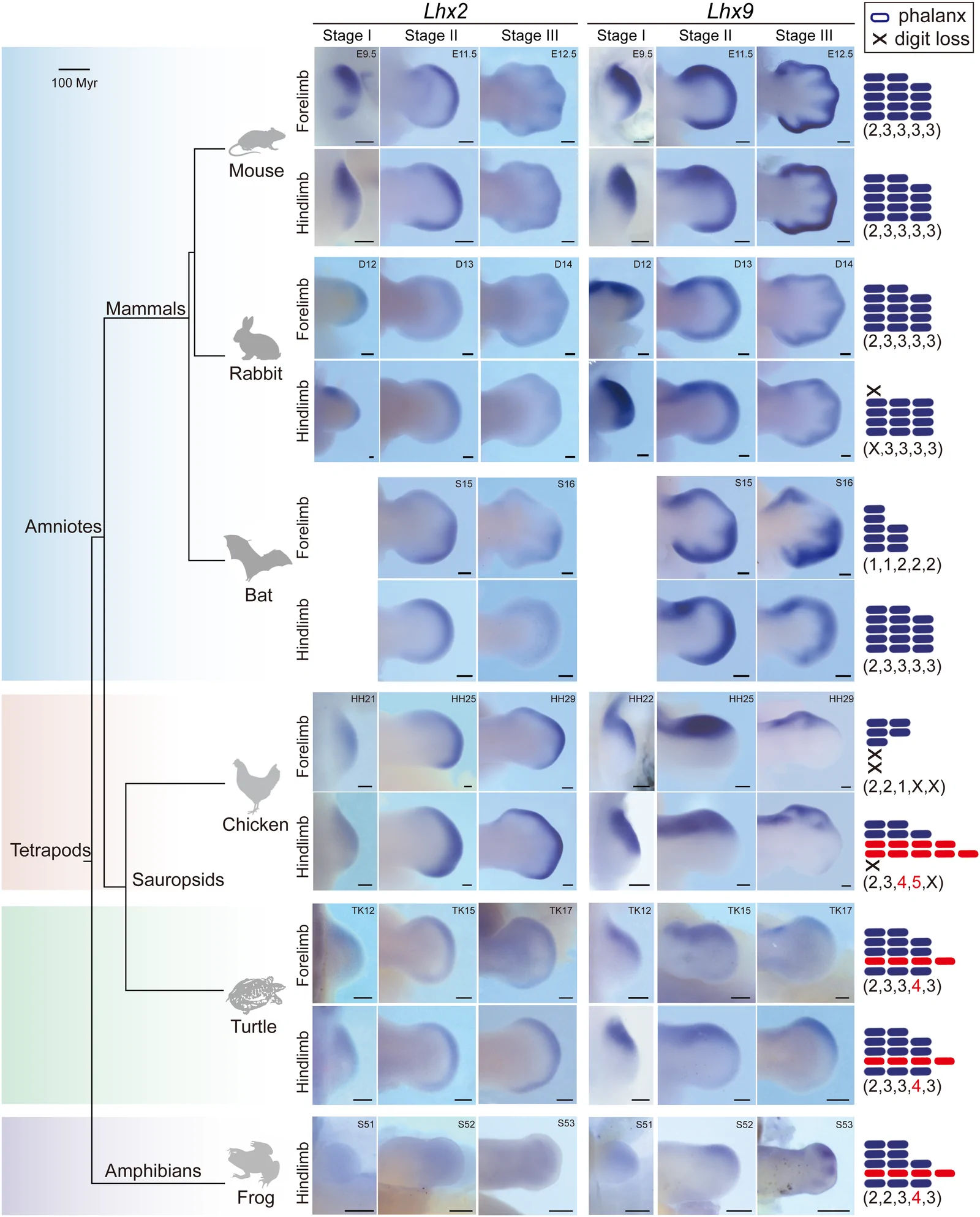
Expression of *Lhx2* and *Lhx9* in the limb buds of six tetrapod species during embryonic development. Bat, Schreibers’ long-fingered bat; turtle, Chinese soft-shelled turtle; frog, African clawed frog. Dorsal view of the limb bud is shown, with anterior to the top and distal to the right. E/D, embryonic day; S, stage; HH, Hamburger-Hamilton stage; TK, Tokita-Kuratani stage. The phalangeal formula is presented in the rightmost column. Digits exhibiting more than three phalanges are highlighted in red. The time tree shows the evolutionary relationships among the six species. Myr, million years. (Scale bar, 200 μm.)

To investigate the spatiotemporal expression patterns of *Lhx2* and *Lhx9* in limbs during embryonic development, we performed whole-mount in situ hybridization (WISH) analyses in six representative tetrapods and compared them at three developmental stages (stage I, early limb bud outgrowth; stage II, autopod outgrowth and early chondrogenic condensation; stage III, the start of sequential phalanx formation). At stage I, *Lhx2* was broadly expressed in the frog hindlimb buds, and was expressed in the distal mesenchymal region of the fore- and hindlimb buds in the amniotes (mouse, rabbit, bat, chicken, and turtle), with higher expression in the anterior region than in the posterior region (Fig. 1). At stages II and III, expression of *Lhx2* was still broad in the frog hindlimb buds but focused on the distal margin of the fore- and hindlimb buds in amniotes, where expression levels were balanced between the anterior and the posterior margins. In contrast, the expression of *Lhx9* exhibited evolutionary divergence. In nonmammalian tetrapods (frog, chicken, and turtle), *Lhx9* was highly restricted to the anterior mesenchyme of the limb buds from stage I to stage III. Critically, in all mammals examined (mouse, rabbit, and bat), *Lhx9* expression expanded from the anterior region to the entire posterior-distal margin of the fore- and hindlimbs and was particularly evident in stages II and III. Reconstruction of the ancestral states (using both ML and parsimony methods) confirmed this divergence: the expression of *Lhx2* was originally dispersed in the whole limb buds and became restricted to the distal region along the apical ectodermal ridge (AER) in the ancestors of amniotes, whereas the expression of *Lhx9* expanded from the anterior region to the posterior-distal region of the limb buds in the ancestors of mammals (*SI Appendix*, Fig. S2). We note that confident statements about the condition in a clade can only be made if the clade is phylogenetically bracketed, or in other words examining at least one taxon from each side of the basal split. For amphibians this would be caecilians (limbless) and frogs or salamanders. For mammals it would be monotremes and therians. For birds it would be palaeognaths and neognaths. Such an approach is not feasible for some of these taxa (e.g., caecilians and monotremes).

To further investigate the expression of *Lhx2* and *Lhx9* during sequential phalanx formation, we performed WISH and cryosection ISH on chicken and mouse hindlimbs at later developmental stages. Skeletal staining confirmed the developmental progression of phalanx formation (*SI Appendix*, Fig. S3). We found that *Lhx2* and *Lhx9* were highly expressed in the distal mesenchymal regions—including PFR—but not in the maturing phalanges (e.g., the growth plate or proliferating chondrocytes). In the mouse, although the overall expression level of *Lhx9* was higher than that of *Lhx2*, their expression patterns were similar and balanced between the anterior and posterior regions (early in the interdigital mesenchyme and later around phalanges). This expression pattern is consistent with findings in humans, where a published study indicated that *Lhx2* was enriched and expressed in distal mesenchymal progenitor populations located at the distal periphery of the human developing limb, which are associated with proliferative digit-patterning regions rather than mature chondrogenic elements (22). In chicken, *Lhx2* expression remained restricted to the distal mesenchymal region along the AER, with higher expression in the posterior regions, whereas *Lhx9* still showed stronger expression in the anterior digits, although weak expression was detected along the posterior digits, highlighting a conserved anterior–posterior asymmetry in sauropsids.

### Functional Divergence of *Lhx2* and *Lhx9* in Regulating Digit Development.

**Fig. 2.**
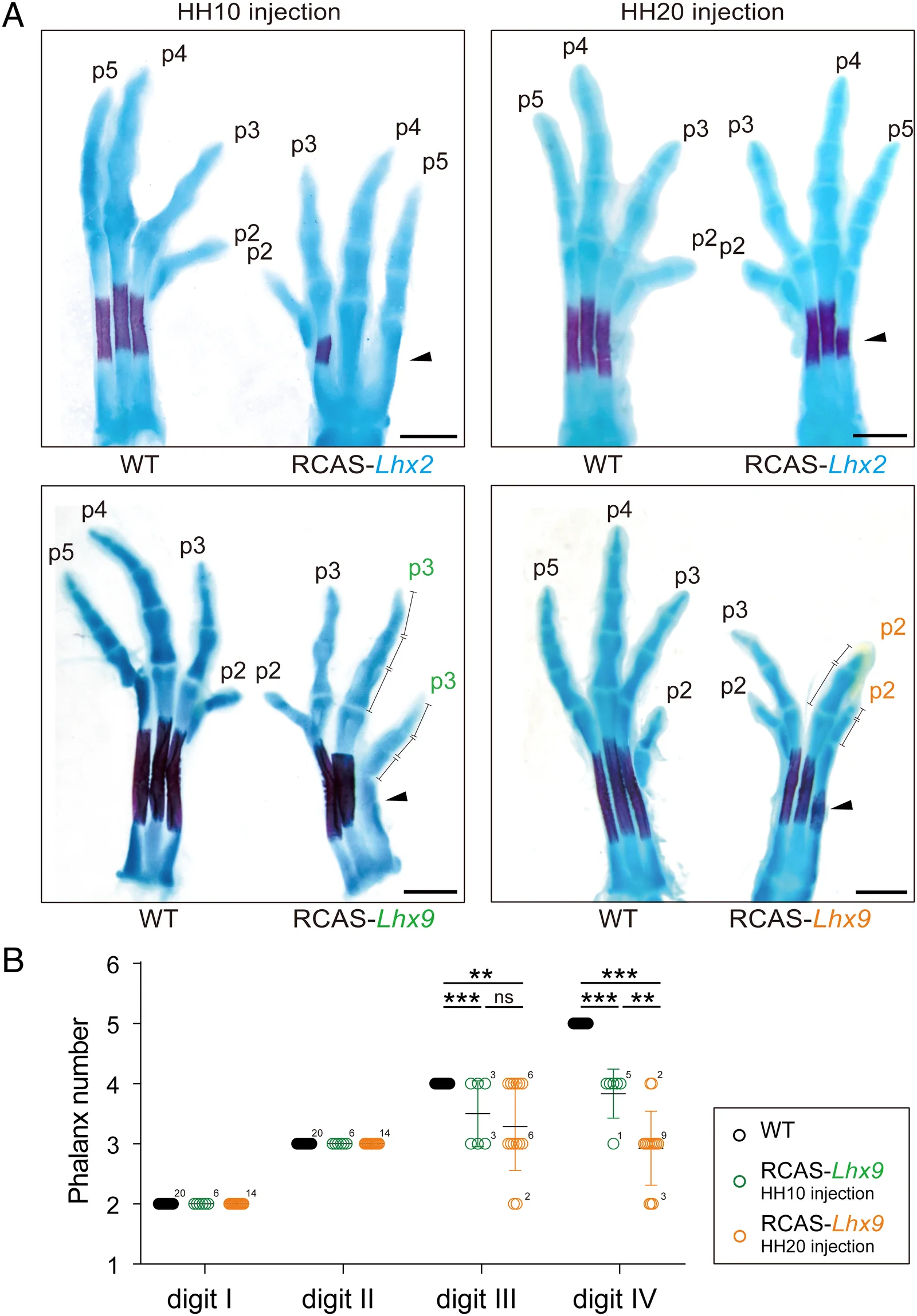
Overexpression effects of *Lhx2* and *Lhx9* in chicken hindlimbs. (*A*) Skeletal staining showing the *Left* (wild-type, WT) and *Right* (*Lhx2/9*-overexpressing) hindlimbs of the same embryo at HH35. The black lines indicate phalanges and the black arrows indicate areas of delayed ossification. p, the number of phalanges; (Scale bar, 1 mm.) (*B*) Quantification of phalanx number in digits I to IV following *Lhx9* overexpression. The number of individuals examined is indicated to the upper right of the dots. Box plots represent the median, quartile partition, range, and individual biological replicates. Student’s *t* test: **P < 0.01, ***P < 0.001, ns, no significance.

To investigate specific functions of *Lhx2* and *Lhx9* in limb development, we overexpressed these two genes in the hindlimbs of chicken embryos using RCAS avian retroviral constructs (injected at HH10 or HH20 and collected at HH35). Control limbs injected with RCAS empty-vector or with RCAS-GFP at HH10 or HH20 were very similar to wild-type limbs (RCAS injected at HH10, *n* = 40; RCAS injected at HH20, *n* = 15; RCAS-GFP injected at HH10, *n* = 45; RCAS-GFP injected at HH20, *n* = 45) (*SI Appendix*, Fig. S4). RT-qPCR and WISH analyses showed that *Lhx2* and *Lhx9* mRNA were significantly up-regulated in the chicken hindlimb buds infected with RCAS-*Lhx2* and RCAS-*Lhx9* confirming that *Lhx2* and *Lhx9* were successfully overexpressed in the chicken embryos (*SI Appendix*, Fig. S5*A*). In chicken hindlimbs overexpressing *Lhx2* or *Lhx9*, we observed obvious skeletal changes in the digits (phalanges and metatarsals), leaving tarsals and proximal bones grossly unremarkable (*SI Appendix*, Fig. S5*B*). Overexpression of *Lhx2* at HH10 or HH20 delayed metatarsal ossification in 48.7% of embryos (37/76; Fig. 2*A*). Similarly, overexpression of *Lhx9* led to delayed metatarsal ossification in 50.6% of embryos (42/83; Fig. 2*A*). Overexpression of both genes significantly shortened the posterior digits III and IV (*SI Appendix*, Fig. S5*C*).

In contrast to *Lhx2*, 47.6% (20/42) of phenotypically abnormal *Lhx9*-overexpressing embryos showed a reduction in phalanx number from 4–5 to 2–3 in the third and fourth digits (Fig. 2*A* and *SI Appendix*, Fig. S5*B*). We next quantified phalanx number digit by digit across all defected limbs. Digits I and II were unchanged in all measured samples, whereas digits III and IV showed clear reductions after *Lhx9* overexpression (Fig. 2*B*). In detail, embryos injected with virus at HH20 possessed as few as two phalanges on digits III to IV (*n* = 14) and those injected with virus at HH10 had as few as three phalanges on digits III to IV (*n* = 6). While phalanx number was reduced in *Lhx9*-overexpressing limbs, we did not observe a significant change in phalangeal width based on statistical analysis (*SI Appendix*, Fig. S5*C*). The phenotypes observed following *Lhx2* overexpression at both HH10 and HH20 were similar, as overexpression at neither time point was able to reduce the number of phalanges (Fig. 2*A* and *SI Appendix*, Fig. S5*B*). These results suggest that overexpression of both *Lhx2* and *Lhx9* reduces digit length, whereas overexpression of *Lhx9* is uniquely associated with a reduction in phalanx number, indicating functional differences between the two genes during limb development.

### mRNA-seq Analysis of Limb Transcriptomes with *Lhx2* and *Lhx9* Overexpression.

**Fig. 3.**
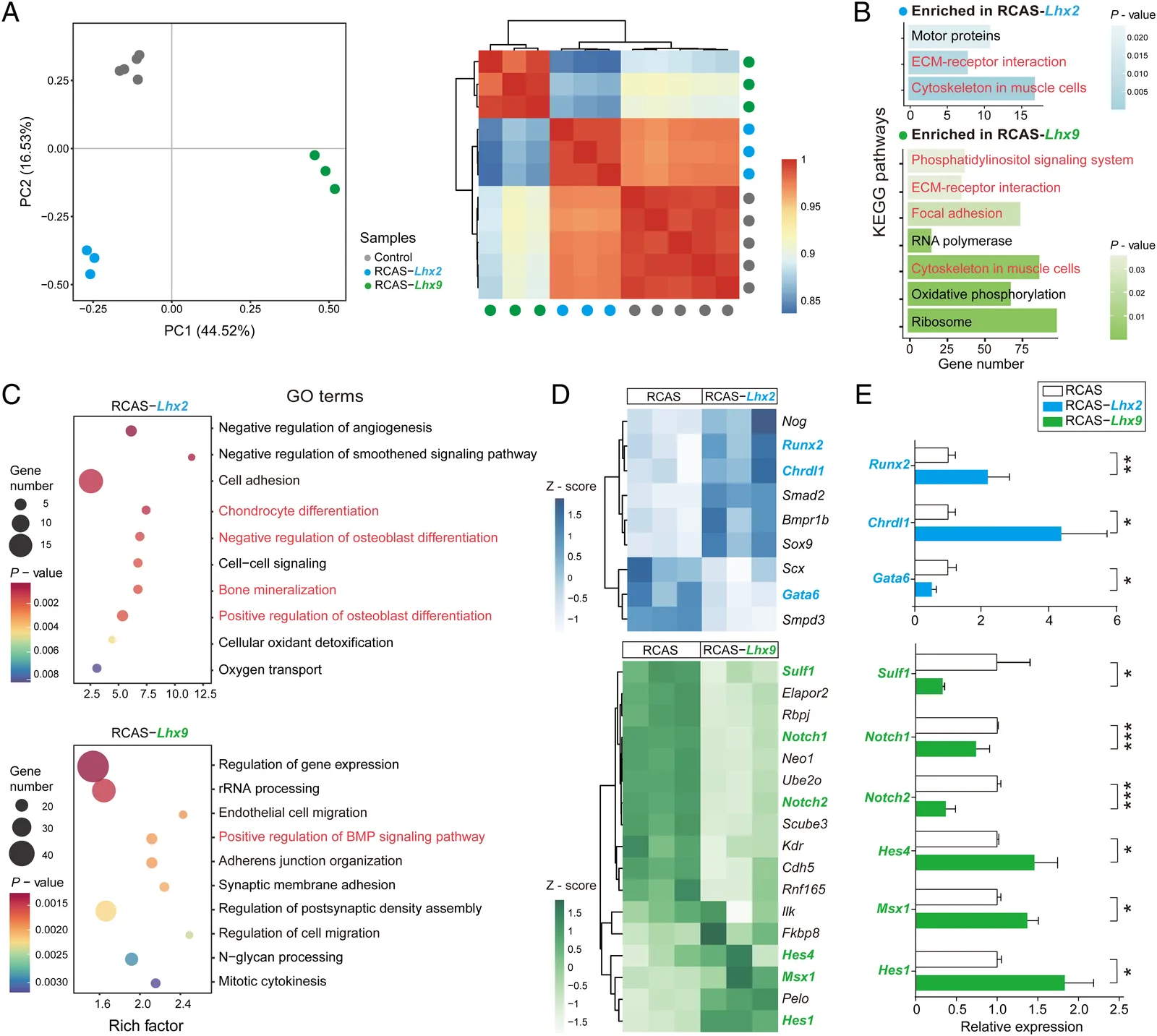
mRNA-seq analyses of chicken hindlimb autopods with *Lhx2* and *Lhx9* overexpression. (*A*) PCA and Spearman correlation analyses of mRNA-seq samples. (*B*) KEGG analysis of DEGs identified following RCAS-*Lhx2* and RCAS-*Lhx9* injection. (*C*) Top 10 terms identified by GO-BP analysis using all DEGs. Terms related to limb development are highlighted in red in *B* and *C*. (*D*) Heatmap of DEGs enriched for the BMP signaling pathway after *Lhx9* and *Lhx2* overexpression. (*E*) RT-qPCR analysis showing the expression levels of DEGs in *D*. *n* = 3 biological replicates. Student’s *t* test: *P < 0.05, **P < 0.01, ***P < 0.001.

To define the molecular pathways that *Lhx2* and *Lhx9* use to influence the regulation of limb development, we performed mRNA-seq on autopod tissue from chicken hindlimbs infected with RCAS-*Lhx2*, RCAS-*Lhx9*, or an RCAS control (*SI Appendix*, Fig. S6). Tissue was collected at HH30, a stage when phalanx formation is initiated, as indicated by alcian blue staining and *Gdf5* expression (*SI Appendix*, Figs. S3 and S6). Principal component analysis (PCA) and Spearman correlation heatmap analysis of the transcriptomic profiles identified three distinctive gene expression clusters, each consistently reproduced across all biological replicates (Fig. 3*A*), confirming the reproducibility of the transcriptional responses. Furthermore, *Lhx9* overexpression appeared to have a stronger impact on the transcriptome than *Lhx2* overexpression (Fig. 3*A*). Indeed, differential expression analysis identified 681 differentially expressed genes (DEGs) after *Lhx2* overexpression and 5,219 DEGs after *Lhx9* overexpression (Dataset S1), with 208 overlapping DEGs (*SI Appendix*, Fig. S7*A*). Gene Ontology-Biological Process (GO-BP) enrichment analysis showed that the overlapping DEGs due to *Lhx2* and *Lhx9* overexpression were significantly enriched in skeletal related terms, such as positive regulation of chondrocyte differentiation and bone mineralization (*SI Appendix*, Fig. S7*B*). Kyoto Encyclopedia of Genes and Genomes (KEGG) pathway enrichment analysis revealed that the overlapping DEGs resulting from *Lhx2* and *Lhx9* overexpression were enriched in the cytoskeleton in muscle cells.

To explore the respective transcriptional properties of *Lhx2* and *Lhx9*, we performed further enrichment analyses on their respective DEGs. KEGG pathway enrichment analysis revealed that DEGs resulting from *Lhx2* overexpression were enriched in several pathways, including ECM–receptor interaction and cytoskeleton in muscle cells (Fig. 3*B*). GO-BP enrichment analysis showed that the DEGs due to *Lhx2* overexpression were significantly enriched in skeletal related terms, such as chondrocyte differentiation, negative regulation of osteoblast differentiation, bone mineralization, and positive regulation of osteoblast differentiation (Fig. 3*C* and Dataset S2). KEGG analysis showed that DEGs that follow *Lhx9* overexpression were significantly enriched in limb development-related pathways, including phosphatidylinositol signaling system, ECM–receptor interaction, focal adhesion, and cytoskeleton in muscle cells (Fig. 3*B*). GO analysis revealed that *Lhx9* overexpression significantly enriched 17 DEGs involved in the positive regulation of the BMP signaling pathway, which is critical for limb skeletal development (vs. 9 DEGs for *Lhx2*; Fig. 3 *C* and *D* and Dataset S2). Nine representative BMP pathway genes from the *Lhx9*- and *Lhx2*-overexpression groups were selected for RT-qPCR validation, and all showed expression trends consistent with the mRNA-seq data (Fig. 3*E*). We also validated four DEGs (*Gli3*, *Alx4*, *Fgf8*, and *Hoxd11*) by WISH and RT-qPCR following *Lhx9* overexpression, all of which are well-established regulators of limb development (6, 23, 24) (*SI Appendix*, Fig. S7*C* and Dataset S1). Notably, the irregular AER pattern observed in *Fgf8* WISH of HH30 control limbs reflects the physiological developmental regression of endogenous *Fgf8* expression at this stage, as previously reported (25, 26), and is unlikely to result from technical artifacts, as all samples were processed in parallel under identical conditions. Consistent with our observation, similar irregular *Fgf8* expression in control limbs at HH31 has also been documented in a previous study (6), indicating that such morphological variability is common at these late limb bud stages. These validation results support the reliability of the transcriptomic analysis.

### Analyses of Lhx2 and Lhx9 Protein Sequences.

To evaluate whether lineage-specific functional divergence of Lhx2 and Lhx9 might be associated with changes in their protein-coding sequences, we compared the amino acid sequences of the two proteins between mammals and sauropsids (*SI Appendix*, Fig. S8*A*). The results showed both proteins shared the same three domains: the LIM zinc-binding 1 (LIM1), LIM zinc-binding 2 (LIM2), and the homeobox domain (HD). There were three amino acid differences between Lhx2 and Lhx9 in LIM1, four differences between the two genes in LIM2, and the HD was identical. AIUPred predicted pronounced intrinsically disordered regions (IDRs) at the N and C termini of the chicken Lhx2 and Lhx9 amino acid sequences, a pattern consistent with the role of IDRs in mediating regulatory functions of transcription factors (*SI Appendix*, Fig. S8*B*). Structural similarities were also observed in the HD and LIM domains, which showed low peaks (below 0.5 for LIM1 and LIM2, and around 0.5 for HD). The conserved ordered domains are flanked by extensive IDRs, which may underlie distinct regulatory functions beyond DNA binding. The functions of IDRs need to be tested experimentally, for example by coimmunoprecipitation (Co-IP) assays to test for interactions with candidate cofactors, and reporter-based functional assays to assess transcriptional activity.

To evaluate whether the functional divergence between Lhx2 and Lhx9 was associated with protein sequence changes, we mapped branch-wise inferred amino acid substitutions across tetrapod lineages (*SI Appendix*, Fig. S9). The results showed that Lhx2 and Lhx9 were generally conserved, with amino acid substitutions in most branches below five. On the ancestral branches of the four major clades (amniotes, mammals, sauropsids, and amphibians), the amino acid substitutions of the two genes were all below 10, except for the ancestors of mammals, which had 27 substitutions in Lhx2. To further investigate the selective pressure experienced by *Lhx2* and *Lhx9* during tetrapod evolution, we performed molecular evolutionary analyses on the coding sequence of these genes across 41 species using PAML (27). From the results of the free-ratio model, we found that the ancestors of mammals had both high synonymous and nonsynonymous substitutions in Lhx2, resulting in an *ω* much lower than 1 (*SI Appendix*, Fig. S9). The results showed that one-ratio models were better than two-ratio models for both *Lhx2* and *Lhx9*, with a shared *ω* ratio of 0.03 (*SI Appendix*, Table S1). Although the Clade model C was significantly better than the null model for both mammalian and sauropsid foregrounds for the two genes, all estimated *ω* values were much less than 1. These results demonstrate that both *Lhx2* and *Lhx9* genes are under strong purifying selection, with no evidence supporting adaptive evolution in mammals or sauropsids.

## Discussion

In this study, we identify *Lhx9*—but not *Lhx2*—as playing a role in reducing the phalanx number of posterior digits. *Lhx9* exhibits a mammalian-specific expression pattern in the posterior region of limb buds. To our knowledge, no other gene has been found to possess both this unique function and specific expression pattern. These results suggest that *Lhx9* is likely a key factor in reducing the phalanx number of digits III to V during mammalian evolution. Furthermore, the conserved anterior expression of *Lhx9* across all tetrapods may also contribute to reducing the phalanx number of digits I to II to three or fewer. *Lhx2* and *Lhx9* exhibit overlapping functions in digit shortening, consistent with their highly conserved DNA binding domains. Conversely, their functional divergence may stem from sequence variations in the effector domains and nondomain regions including IDRs (28, 29).

Our ISH results reveal high expression of *Lhx2* and *Lhx9* in the distal limb mesenchyme, a key mesenchymal region that regulates digit patterning (22). Our findings are in accordance with previously published single-cell RNA-sequencing (scRNA-seq) data. In the mouse limb, *Lhx2* and *Lhx9* are reported to be coexpressed in limb naive progenitors, which progressively transition into autopodial progenitors over several days and are associated with autopod patterning (30). During the early stage of sequential phalanx formation, scRNA-seq datasets from chicken, mouse, and bat further reveal that *Lhx2* and *Lhx9* are expressed in distal mesenchymal and marginal cell populations associated with autopod outgrowth and phalanx formation (31, 32). In the early emu limb bud, scRNA-seq analysis shows that *Lhx9* is enriched in the anterior cell population (33), consistent with its previously observed anterior bias in chicken mRNA-seq and ISH data (20). Together, these results underscore a spatial distinction between the two paralogs: while both are expressed in the distal mesenchyme, *Lhx9* shows an anterior bias in the developing avian limb. The results of this study, supported by previous studies, clearly indicate that *Lhx2* and *Lhx9* have an important role in progenitor cell maintenance during early limb bud emergence that may be distinct from their roles during later stages of digit morphogenesis—i.e., only *Lhx9* is capable of regulating phalanx number. Notably, *Lhx9* overexpression caused a more severe reduction in phalanx number at HH20 than at HH10. The reason for this difference is unknown; one possibility might be compensation by an unknown factor, but this remains to be evaluated.

Through transcriptomic analysis, we further revealed both shared and divergent DEGs and pathways regulated by *Lhx2* and *Lhx9*. *Lhx9* overexpression generated considerably more DEGs than *Lhx2* overexpression, likely reflecting its specific role in phalanx development and indicating its broader transcriptional influence and greater biological impact. Despite this difference in transcriptional breadth, both genes converge on key developmental pathways, including cytoskeleton in muscle cells and ECM–receptor interaction—both of which are known to play pivotal roles in chondrogenesis and skeletal development (34, 35). Moreover, *Lhx2* and *Lhx9* coregulate chondrocyte differentiation and bone mineralization and share common target DEGs such as *Sox9* and *Bmpr1b*, which are expressed within the developing digit ray and have established roles in skeletal development (5, 36–38). Together, these findings suggest that both paralogs participate in a common molecular framework underlying distal limb skeletal development.

Despite the above overlapping DEGs and pathways, the gene-specific transcriptional signatures differed markedly. Among the DEGs specifically altered by *Lhx9* overexpression, changes in *Gli3*, *Alx4*, *Fgf8*, and *Hoxd11* may reflect perturbation of regulatory programs involved in distal limb patterning and growth (6, 39–41); however, whether these changes contribute to the reduced phalanx number and digit ray shortening remains to be determined. The *Lhx9* overexpression dataset also showed a strong association with the BMP signaling pathway, including six genes directly related to phalangeal morphogenesis—*Hes1, Hes4, Msx1, Notch1, Notch2,* and *Sulf1* (42–47). Among these, *Hes1*, *Hes4*, and *Msx1* were upregulated, whereas *Notch1*, *Notch2*, and *Sulf1* were downregulated. Increased expression of *Hes1* and *Hes4* in mouse and chicken limb models was found to delay chondrogenesis and shorten skeletal segments (42, 45). The upregulation of *Msx1*, a marker associated with claw development, is consistent with comparative expression patterns linked to variation in phalanx number among amniotes (47). Loss-of-function mutations in *Notch1* and *Notch2* have been clinically associated with terminal limb abnormalities, including shortened terminal phalanges, although the mechanistic basis of this association remains unclear (44, 46). Furthermore, *Sulf1* is critical for regulating multiple stages of cartilage development and joint formation, as its deletion in humans results in distal limb defects and shortened digits (43). Taken together, the concerted upregulation of *Hes1*, *Hes4*, and *Msx1* and downregulation of *Notch1*, *Notch2*, and *Sulf1* by *Lhx9* overexpression may collectively contribute to the observed delay in phalanx formation and reduction in phalanx number. To further evaluate how *Lhx9* modulates phalanx number, future studies could focus on the phalanx-forming regions at earlier stages (e.g., HH28–29) to capture the earliest transcriptional changes that precede the overt morphological phenotype.

*Lhx2*-specifically modulated DEGs are enriched in processes associated with skeletal development and ossification, consistent with the *Lhx2* phenotype of delayed ossification and shortened autopods without phalanx loss. In the *Lhx2*-overexpression dataset, the most strikingly enriched biological processes are related to chondrocyte differentiation, osteoblast differentiation, and bone mineralization, suggesting that *Lhx2* primarily modulates skeletal growth and differentiation. Correspondingly, BMP-associated genes altered by *Lhx2* include several regulators involved in limb skeletal development and patterning. Among them, *Runx2* and *Chrdl1* were significantly upregulated, whereas *Gata6* was downregulated. Upregulation of *Runx2* results in brachydactyly in humans (48), and overexpression of *Chrdl1* causes loss of skeletal elements in the chicken hindlimb (49)—both consistent with the observed changes in our *Lhx2*-overexpression dataset. In addition, *Gata6* is involved in anterior–posterior patterning of the autopod, as its absence in mice induces the formation of an extra anterior digit together with associated tarsal and metatarsal elements (50), while the potential relationship between its downregulation and the shortened autopod phenotype induced by *Lhx2* overexpression remains to be investigated. Together, these results suggest that *Lhx2* acts primarily to regulate limb skeletal growth rather than to determine phalanx number.

The coding sequences of *Lhx2* and *Lhx9* are both highly conserved across tetrapods, pointing to conserved functions, yet their expression patterns have evolved differently (*SI Appendix*, Fig. S2). *Lhx2* expression narrowed from the entire to the distal limb bud in the amniote ancestor, while *Lhx9* expression expanded anteriorly to posteriorly in the mammal ancestor. These results reflect subfunctionalization following an early vertebrate gene duplication event, in which the paralogs *Lhx2* and *Lhx9* first diverged in function, and subsequently in their expression patterns (13, 51, 52). Thus, both functional and regulatory differentiation contributed to the evolution of morphological diversity in digits (53, 54).

The role of *Lhx9* in regulating digit morphology is consistent with the hypothesis that morphological evolution often results from changes in gene expression rather than coding mutations (55). In the forelimbs of bats, for example, we propose that phalanx reduction is regulated by *Lhx9* overexpression, whereas previous studies have shown that phalangeal elongation is controlled by enhanced expression of *Hoxd9–13* genes—along with other genes governing interdigital membranes—thereby shaping the skeletal adaptations for flight (31, 56, 57). Our transcriptomic analysis reveals that *Lhx9* overexpression induces only a significant increase in *Hoxd11* (*SI Appendix*, Fig. S7*C*), with no significant changes in other *Hoxd* genes (Dataset S1). This pattern underscores that *Lhx9* primarily governs phalanx reduction rather than contributing to phalangeal elongation, a process controlled by a bat-specific enhancer (58). In contrast to bats, phalanx reduction in the posterior digits of avian forelimbs likely arises from a distinct evolutionary digit-loss program (23, 59)—potentially involving *Socs2* (60)—rendering posterior phalanx reduction independent of *Lhx9*. Future studies are needed to determine why the expression pattern of *Lhx2* changed in the ancestors of amniotes. In addition, cetaceans exhibit hyperphalangy in their flipper, potentially involving altered expression of *Lhx9*, however, this will be difficult to determine as it would require embryonic samples from these species.

In conclusion, our study provides developmental genetic insights into the evolution of limb morphology across tetrapods. *Lhx2* and *Lhx9*, a pair of paralogous genes derived from an early vertebrate genome duplication (61), underwent functional divergence postduplication: *Lhx2* retained the conserved role in regulating ossification timing and growth of distal skeletal elements, whereas *Lhx9* acquired a function of reducing the phalanx number. Expansion of *Lhx9* expression in the posterior mesenchyme of mammalian autopods may represent an evolutionary shift in expression regulation, resulting in a reduction of phalanx number in the posterior digits and contributing to the establishment of the “2-3-3-3-3” phalangeal formula. Such regulatory innovations may be related to the mechanical demands of adapting to terrestrial locomotion (62). Our study not only exemplifies how expression evolution of duplicated genes with functional divergence can drive morphological diversification but also provides a foundation for analyzing the mechanisms of digit structure evolution in tetrapods.

## Materials and Methods

### Sample Collection.

Embryos from six species of tetrapod, including African clawed frog (*Xenopus laevis*, S51–53), chicken (*Gallus gallus*, HH22–35), Chinese soft-shelled turtle (*Pelodiscus sinensis*, TK12–18), mouse (*Mus musculus*, E9.5–14), rabbit (*Oryctolagus cuniculus*, gestational day D12–14), and bat (*Miniopterus schreibersii,* S15–16), were collected for sequence acquisition and ISH. Chicken embryos were also used for overexpression experiments and mRNA-seq analyses. Staging criteria for these species were from previous studies (63–67). The procedures for embryo collection, incubation, and maintenance of chicken, mice, bats, turtles, and frogs were conducted as described in our previous studies (56, 68, 69). Rabbits were purchased from Liaoning Changsheng Biological Company (Shenyang, China).

### Ancestral State Reconstruction.

Phylogenetic trees derived from TimeTree (70–74) were visualized with iTOL (75). To reconstruct phalangeal patterns of the ancestral tetrapods, we used the maximum likelihood (ML) method in Mesquite 4.01 (76). To avoid disturbing reconstruction, lineages that are known to have lost limbs were excluded from the ancestral character state reconstruction, including snakes, caecilians, and whales (77, 78). For extinct species, we recorded the characters that had been documented in previous studies (3, 79–83). The reconstruction of ancestral expression patterns for *Lhx2* and *Lhx9* used ML and parsimony methods (76). Each expression pattern was assigned a numerical code to facilitate the reconstruction of the ancestral states.

### In Situ Hybridization.

Whole-mount and cryosection ISH assays were performed as previously described (84, 85). Probe templates were amplified by PCR with primers listed in *SI Appendix*, Table S2. For WISH, embryos were hybridized with 1 to 4 μg/mL DIG-labeled RNA probes overnight at 65 °C. For cryosection ISH, 13 μm cryosections were air-dried for 15 min and hybridized with 1 μg/mL probes overnight at 70 °C. Signals in both assays were developed using NBT/BCIP (Roche).

### Skeletal Staining.

Skeletal staining was performed according to a previous study (86). Briefly, dissected embryonic limbs were fixed in 95% ethanol, incubated in acetone, then stained with Alcian blue and Alizarin red to visualize cartilage and bone, and cleared in 1% potassium hydroxide. For cartilage-only staining, samples were fixed in Bouin’s solution, stained with Alcian blue, dehydrated in methanol, and cleared in benzyl alcohol/benzyl benzoate.

### Gene Overexpression.

The whole coding sequences of chicken *Lhx2* and *Lhx9* were amplified and cloned into the *Not* I and *Cla* I sites of RCAS (a generous gift from Clifford J. Tabin). Primer sequences and coding sequence accession numbers are provided in *SI Appendix,* Tables S3 and S4. The RCAS virus was generated and collected as previously described (87), and microinjection was performed following an established protocol (88). In this study, 0.2 to 0.3 μL of viral solution was injected into the presumptive hindlimb LPM region at HH10 and the posterior region of hindlimb buds at HH20 using an Eppendorf microinjection system (Hamburg, Germany). To validate the phenotypic results of overexpression, the *Lhx2* and *Lhx9* genes were overexpressed using RCAS viruses prepared in three independent batches. We validated *Lhx2* and *Lhx9* overexpression with three biological replicates using WISH. Additionally, we used RT-qPCR for validation with three biological and three technical replicates.

### RT-qPCR.

RT-qPCR was performed as described in our previous study (68), using primers listed in *SI Appendix*, Table S5.

### mRNA-seq and Data Analysis.

Chicken hindlimb autopods infected with RCAS-*Lhx2*, RCAS-*Lhx9*, or RCAS control were collected at HH30 after HH10 injection, with three to five biological replicates per group (*SI Appendix*, Fig. S5*D*). RNA extraction, library construction, sequencing, read mapping, data normalization, differential expression, and GO/KEGG enrichment analyses were performed as described in our previous study (68). Reads were mapped to the Ensembl *Gallus gallus* reference genome (bGalGal1. mat. broiler. GRCg7b). Genes with adjusted *P* < 0.05 and |log2fold change| ≥ 1 were defined as differentially expressed genes (DEGs). For enrichment analyses, GO terms with *P* < 0.01 and KEGG pathways with *P* < 0.05 were considered significantly enriched. GO and KEGG enrichment visualizations were generated using OmicStudio (https://www.omicstudio.cn/tool) and Hiplot Pro (https://hiplot.com.cn/), respectively.

### Sequence Alignment and Protein Sequence Disorder Prediction.

Whole coding sequences of *Lhx2* and *Lhx9* were obtained from the NCBI genome database (*SI Appendix*, Table S3). The amino acid sequences translated from the coding sequences were aligned using ClustalW implemented in MEGA11 (89, 90). Three domains (LIM zinc-binding 1, LIM zinc-binding 2, and the homeobox domain) were identified by UniProt (https://www.uniprot.org/) (91). Intrinsically disordered regions (IDRs) of the chicken Lhx2 and Lhx9 protein sequences were predicted using the AIUPred (https://iupred2a.elte.hu/) (92), an enhanced version of the original IUPred disorder prediction method, where higher values correspond to a higher probability of disorder.

### Molecular Evolution Analyses.

To test whether *Lhx2* and *Lhx9* experienced adaptive evolution in tetrapods, we estimated the selective pressures acting on the coding sequences of 41 species, including amphibians, reptiles, birds, and mammals, using the CODEML program of PAML X (27). *Lhx2* and *Lhx9* sequences were retrieved from the GenBank database (see accession numbers in *SI Appendix*, Table S3) and aligned using ClustalW implemented in MEGA11 (89, 90). The best-fitting amino acid substitution models (Dayhoff + G for Lhx2 and JTT + G for Lhx9) were selected in ProtTest 3 (93), and phylogenetic trees were derived from TimeTree (70–74) and visualized with iTOL (75).

### Quantification and Statistical Analyses.

Digit length was measured from the midpoint of the digit base to the tip. The proximal phalanx width was measured at the midpoint of the proximal phalanx. Data were analyzed using two-tailed unpaired Student’s *t* test, as indicated in figure legends.

### Study Approval.

All animal procedures were conducted according to the protocol approved by the Institutional Animal Care and Use Committee of Shenyang Agricultural University (approval ID: 202106038).

## Supplementary Material

Appendix 01 (PDF)

Dataset S01 (XLSX)

Dataset S02 (XLSX)

## Data Availability

The mRNA-seq data generated in this study have been deposited in the Gene Expression Omnibus (GEO) with accession numbers GSE307306 and GSE307372 (94, 95). All other data are included in the manuscript and/or supporting information.
