# Supplementary material for: Lhx9 specifically modulates phalanx number, whereas Lhx2 constrains digit length in tetrapods
Source: Proc Natl Acad Sci U S A. 2026 Oct 20;123(43):e2605261123. doi: 10.1073/pnas.2605261123 (PMC13715784; doi:10.1073/pnas.2605261123)
Supplement: Supplementary file 1 [file pnas.2605261123.sapp.pdf]

Supporting Information for

Lhx9 specifically modulates phalanx number, whereas *Lhx2* constrains digit length in tetrapods

Yining Tang[a,1], Shibin Bai[a,b,1], Shanshan Li[a,b,1], Ying Mao[a], Wanyi Dang[a], Huiling Xue[a], Songyang Shang[a], Ziqiu Jia[a], Xiaoping Li[a], Yanlei Liu[a], Xiaona Xu[a], David M. Irwin[c], Junpeng Zhang[a], Shuyi Zhang[a] and Zhe Wang[a,2]

Author affiliations: [a]College of Animal Science and Veterinary Medicine, Shenyang Agricultural University, Shenyang 110866, China; [b]School of Basic Medical Science, Chengde Medical University, Chengde, 067000, Hebei Province, China; and [c]Department of Laboratory Medicine and Pathobiology, University of Toronto, Toronto, Ontario M5S 1A8, Canada

[1]Y.T., S.B., and S.L. contributed equally to this work.
[2]**Corresponding author:** Zhe Wang
Email: zwang@syau.edu.cn

This PDF file includes:

Figures S1 to S9
Tables S1 to S5
Legends for Datasets S1 to S2
SI References

Other supporting materials for this manuscript include the following:

Datasets S1 to S2

Figures

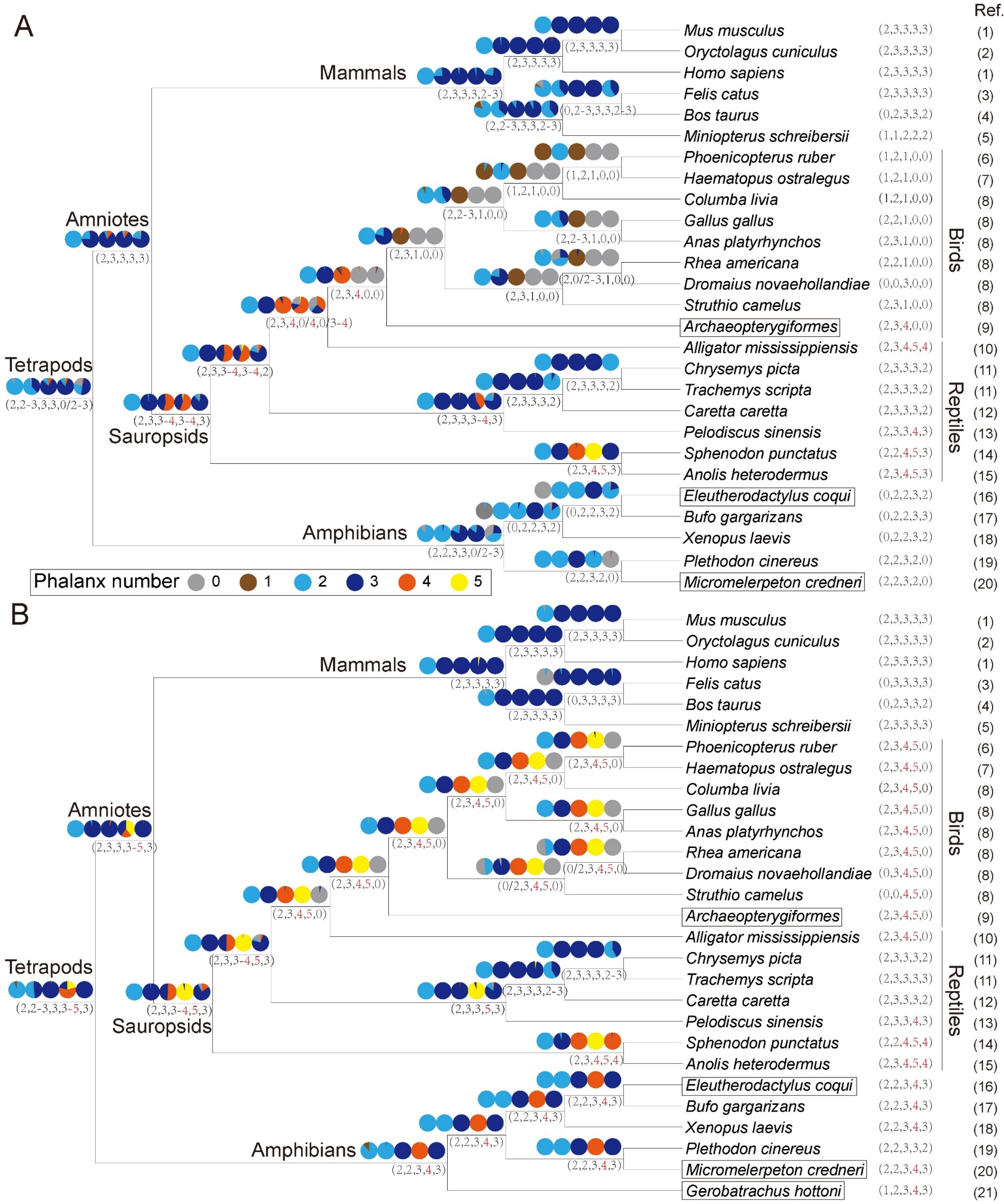

Fig. S1. Reconstruction of phalangeal patterns in tetrapods. A, Forelimbs. B, Hindlimbs. Phalangeal patterns are shown on the branches or after species names. Numbers follow the format: (phalanx number of digit I, digit II, digit III, digit IV, and digit V). Phalangeal counts exceeding three are highlighted in red. Lineages known to have lost either fore- or hindlimbs (e.g., snakes, caecilians, and cetaceans) were excluded from the ancestral character state reconstructions. The probability pie charts and values above and below each branch denote maximum likelihood ancestral state reconstructions. Names of fossil species are in boxes. Ref. (1-21), references.

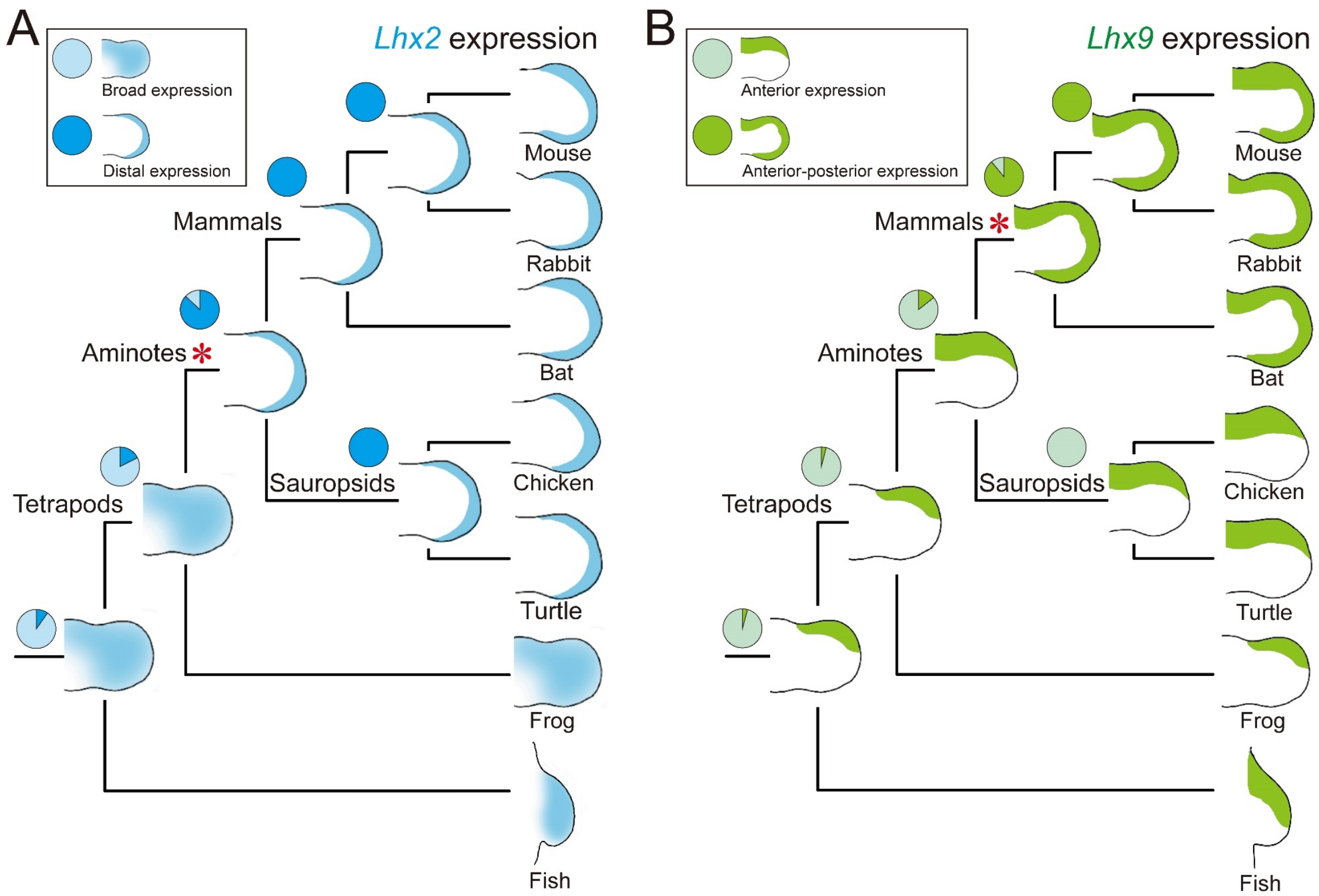

Fig. S2. Comparative schematic of *Lhx2* and *Lhx9* expression patterns in limb and fin evolution. Ancestral expression patterns of *Lhx2* (A) and *Lhx9* (B) were reconstructed using data from this study and from fish (22, 23), and both maximum likelihood (ML) and parsimony methods yielded identical results. Probability pie charts above each branch represent maximum likelihood reconstruction results. Red asterisks indicate key divergence nodes in the evolution of *Lhx2* and *Lhx9* expression. Expression data are available only for pectoral fins in fishes; all other examined species display hindlimb expression patterns. Given that both *Lhx2* and *Lhx9* exhibited similar expression patterns in fore- and hindlimbs, we inferred that the expression pattern of each gene in the pectoral and pelvic fins of fishes is also similar, and thus used the available pectoral fin data to reconstruct gene expression evolution. The limb bud is shown in dorsal view, with anterior to the top and distal to the right.

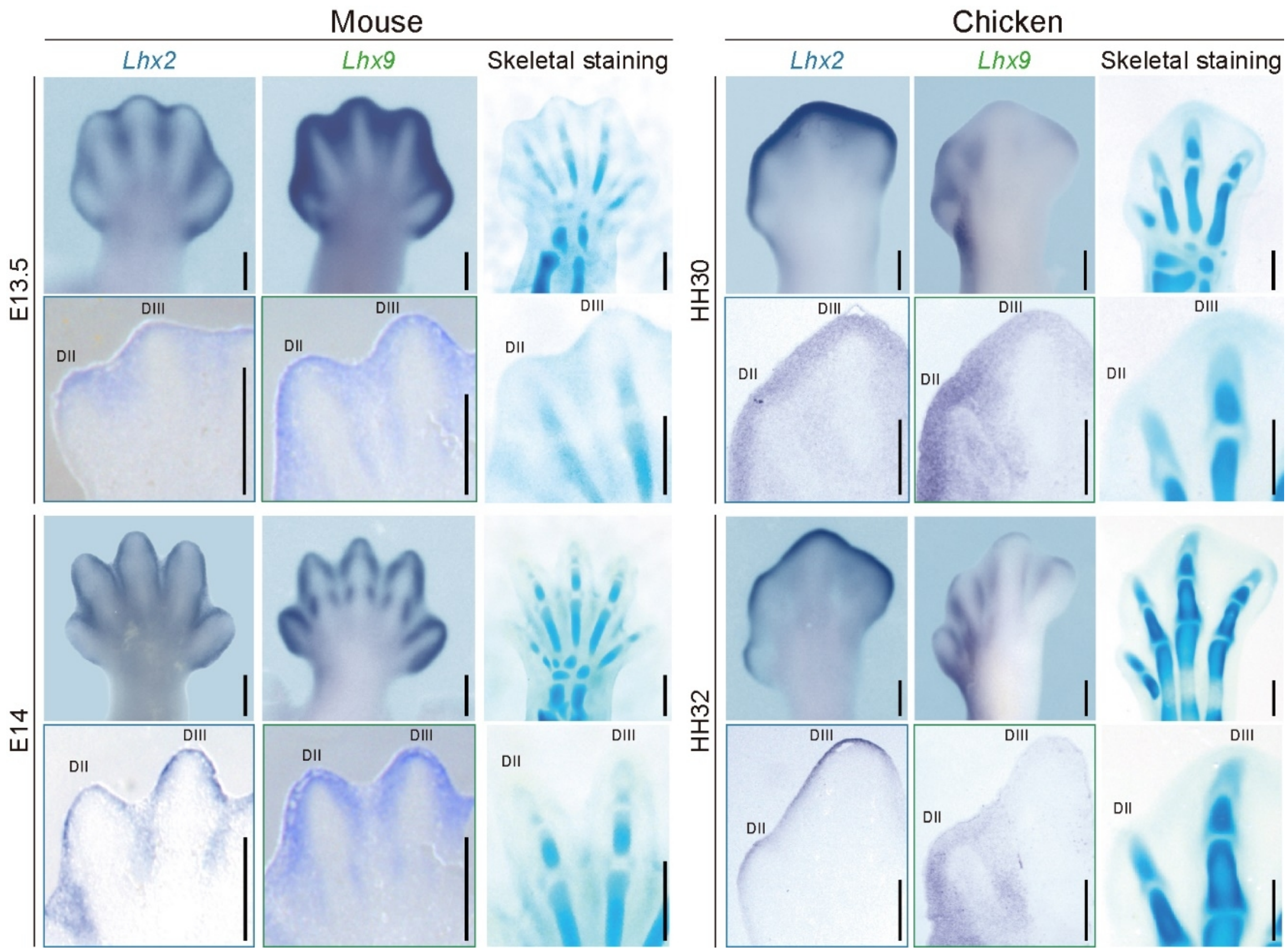

Fig. S3. Expression of *Lhx2* and *Lhx9* in mouse and chicken hindlimbs during digit elongation. Dorsal view of the limb bud is shown, with anterior to the left and distal to the top. Cryosection ISH images are boxed. DII/III, digit II/III. Bars = 500 µm.

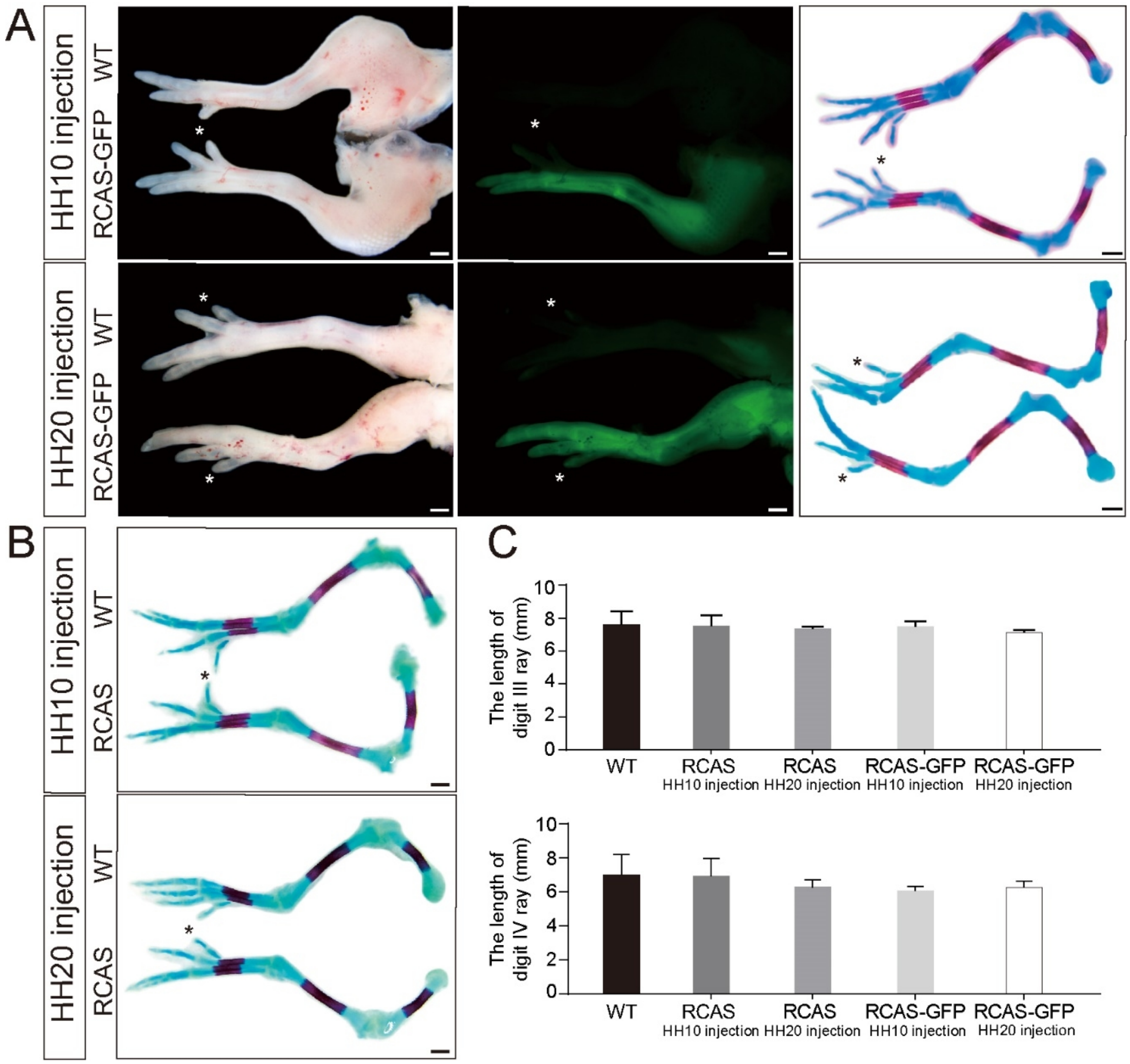

Fig. S4. Phenotypic analysis of chicken control limbs injected with RCAS or RCAS-GFP at HH10 or HH20. A–B, Bright-field, fluorescence, and skeletal staining images. Asterisks indicate digit I. Bars = 1 mm. C, Quantification of digits III and IV in wild-type limbs and limbs injected with RCAS or RCAS-GFP at HH10 or HH20 and examined at HH35. Data are shown as mean ± SEM (n = 6 per group), with no significant difference between the groups. Student's t-test, $P > 0.05$.

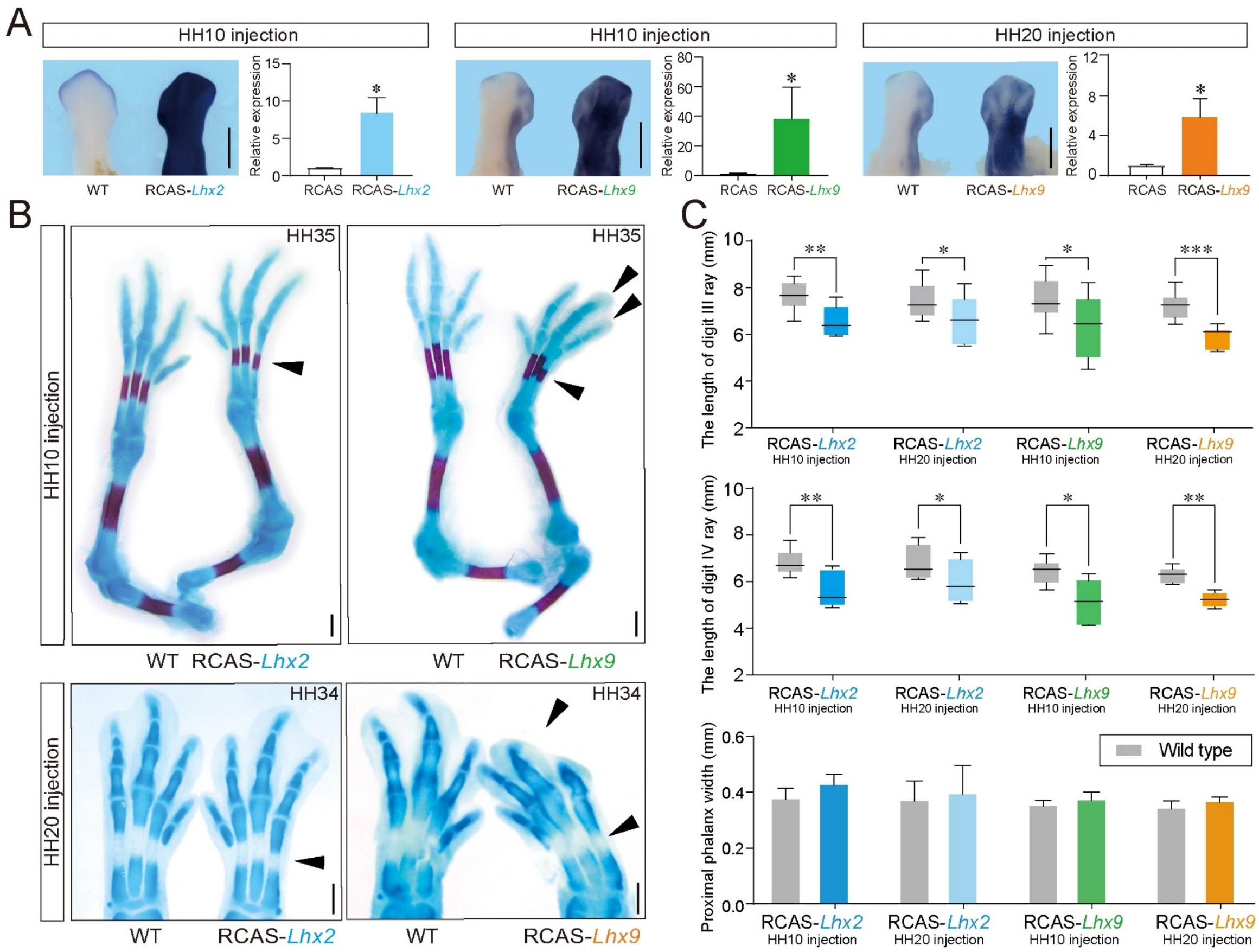

Fig. S5. Analyses of gene expression and skeletal morphology following *Lhx2* and *Lhx9* overexpression in chick hindlimbs. A, Validation of *Lhx2* and *Lhx9* overexpression by WISH at HH28 and RT-qPCR at HH30. Bars = 500 μm. B, Skeletal staining. Alcian blue and Alizarin red staining at HH35, and single Alcian blue staining at HH34. Arrowheads indicate abnormal skeletal elements. Bars = 1 mm. C, Quantification of digit ray length and the proximal phalangeal width in wild-type, *Lhx2- and Lhx9*-overexpressing limbs at HH35. A digit ray comprises the phalanges and the corresponding metatarsal. We measured the lengths of digit rays III and IV, as well as the proximal phalangeal width of digit ray III. Nine embryos were used per group. No significant differences were detected among overexpression groups ($P > 0.05$). The images in A and B show the dorsal view, with distal to the top and digit I placed medially. Student's *t*-test, ***$P < 0.001$, *$P < 0.05$, **$P < 0.01$.

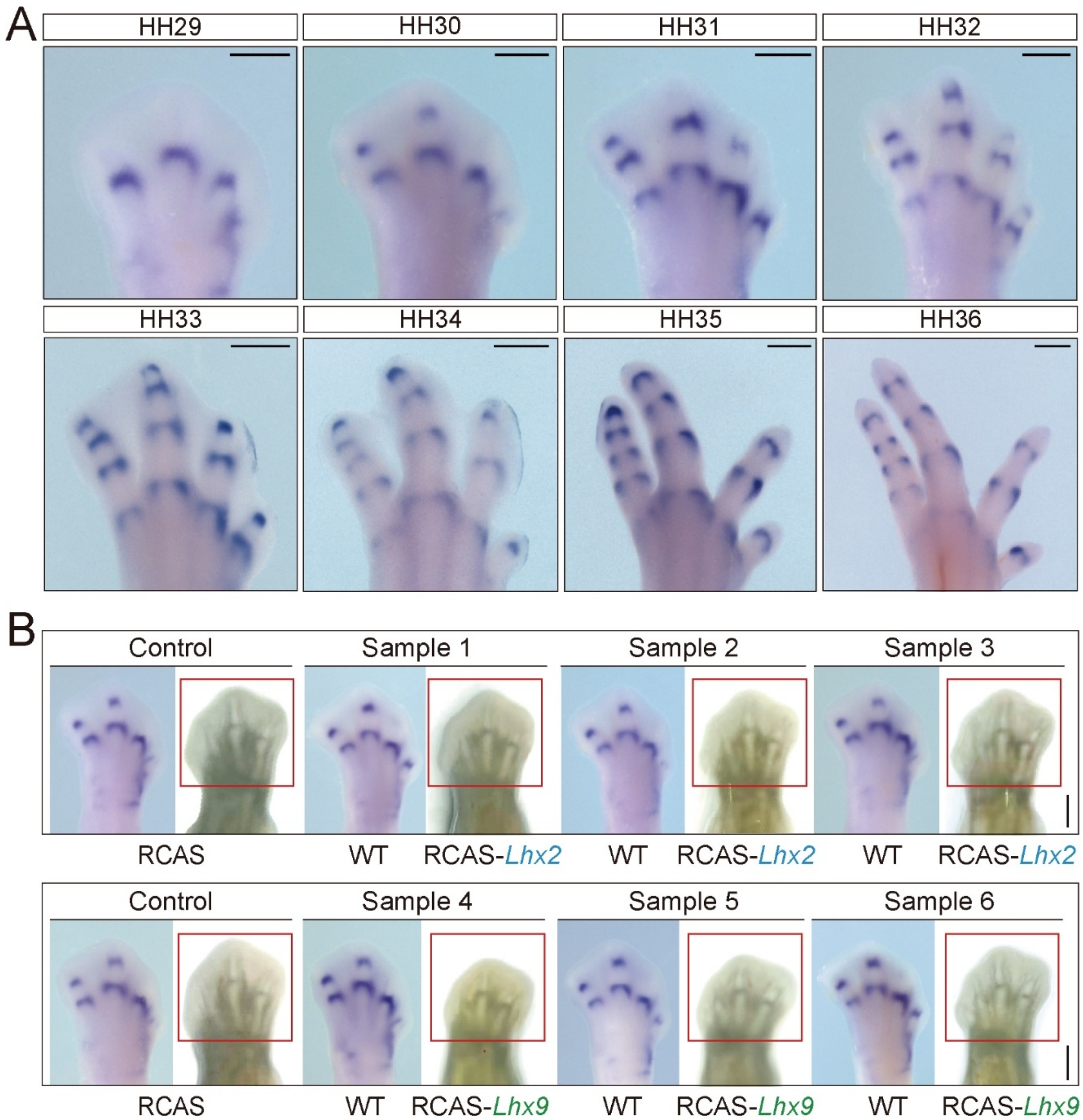

Fig. S6. *Gdf5* expression during phalanx formation and RNA-seq sampling strategy. A, Expression of *Gdf5* in the developing phalanx-forming region of chicken hindlimbs. Dorsal views are shown with distal to the top and digit I to the right. HH, Hamburger–Hamilton stage. B, RNA-seq sampling. Red lines indicate the dissection strategy for collecting mRNA-seq samples from RCAS-*Lhx2*- or RCAS-*Lhx9*-infected hindlimbs. *Gdf5* WISH is shown on the contralateral control limbs. Dorsal views are shown with distal to the top and digit I oriented medially. Scale bars in A and B, 500 μm.

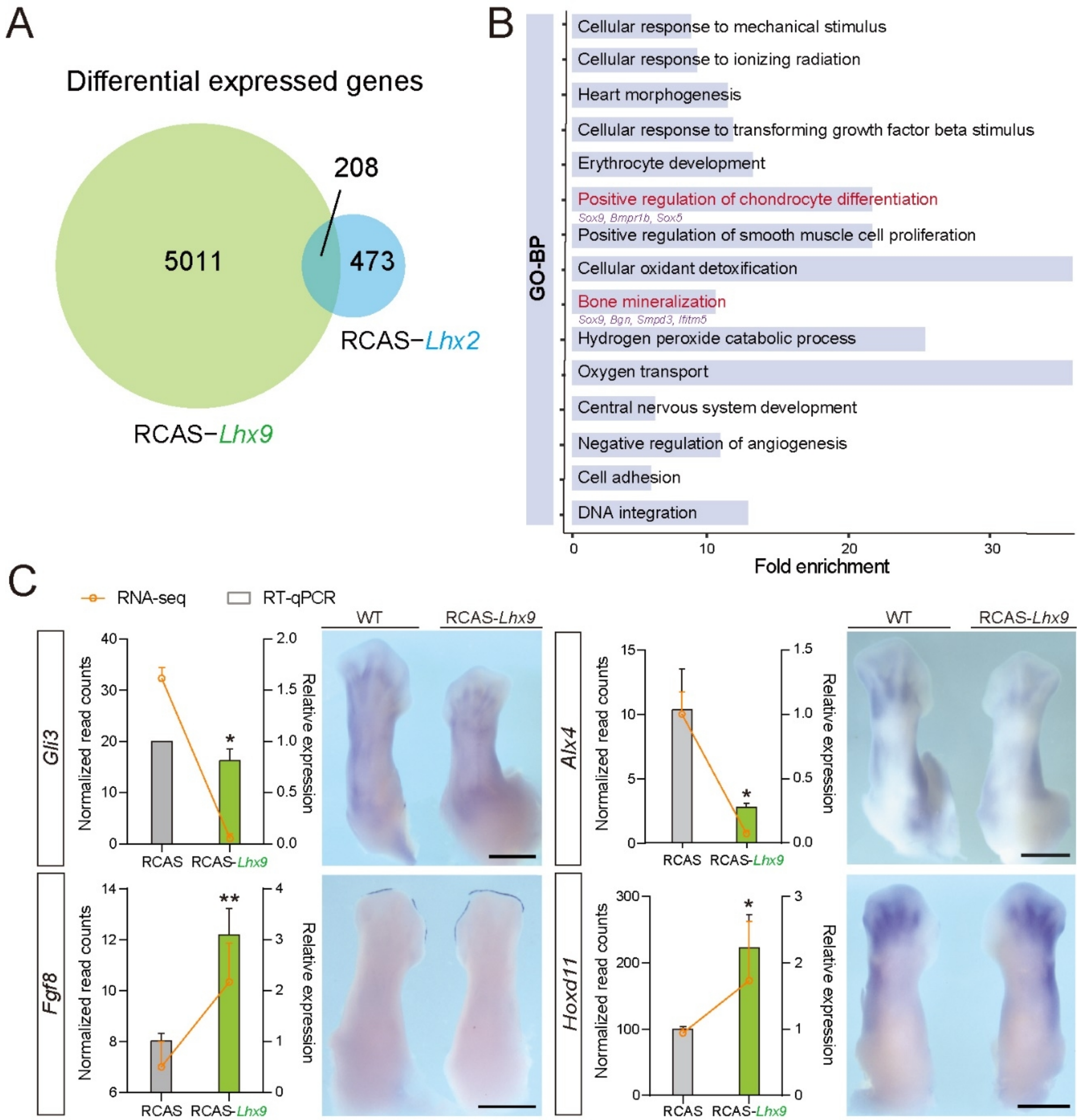

Fig. S7. Analyses of DEGs in *Lhx2*- and *Lhx9*-overexpressing limbs. A, Venn diagram of DEGs from *Lhx2*- and *Lhx9*-overexpressing limbs. B, Top 15 pathways from GO-BP enrichment analysis of the 208 overlapping DEGs. Terms associated with limb development are highlighted in red, and the corresponding DEGs are listed below. C, RT-qPCR and WISH validation of four DEGs following *Lhx9* overexpression at HH30 (n = 3 per group). Student's t-test: $^{*}P < 0.05$, $^{**}P < 0.01$. Dorsal views are shown with distal to the top and digit I oriented medially. Scale bars, 500 μm.

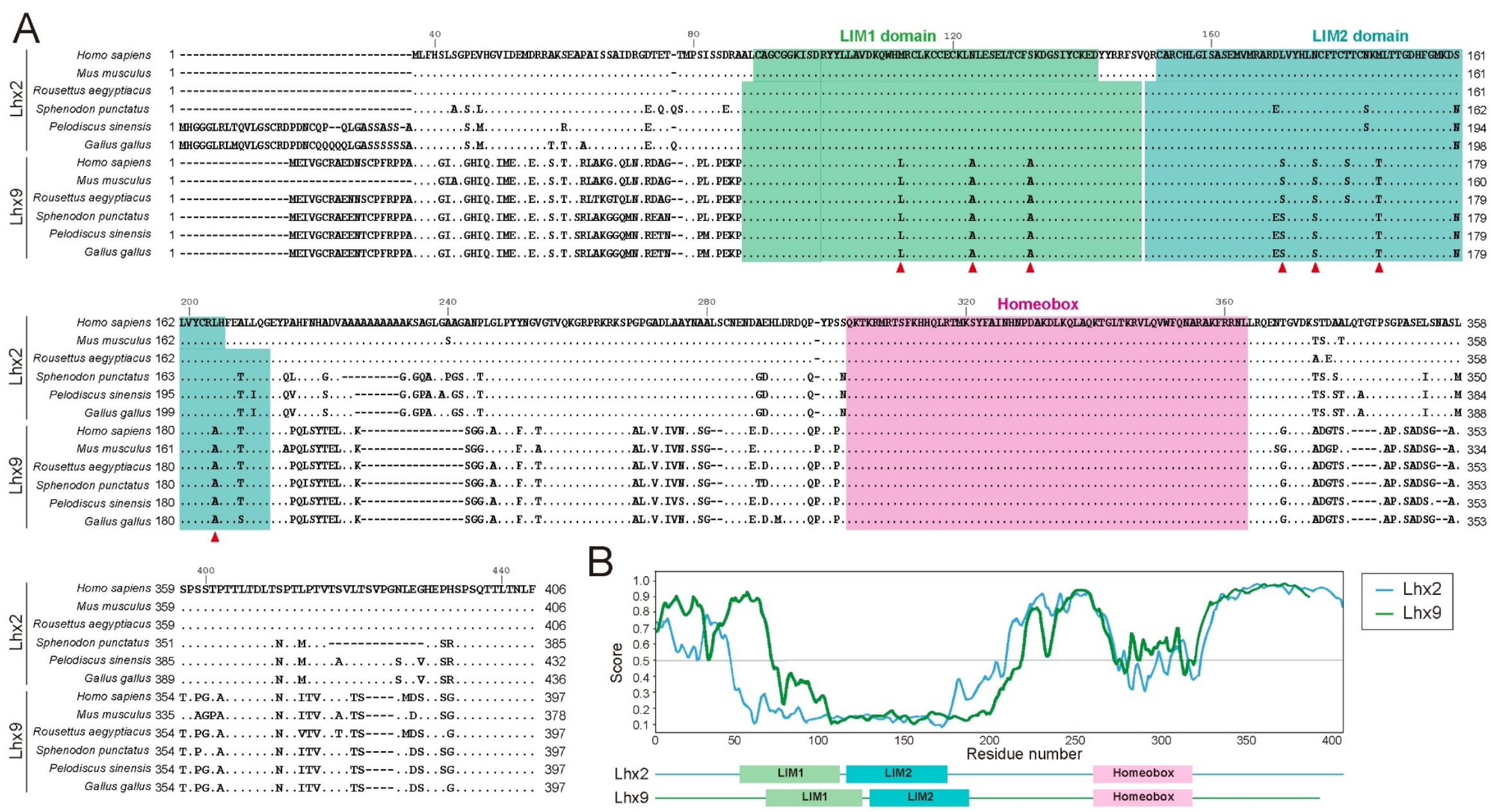

Fig. S8. Sequence alignment and disorder prediction for Lhx2 and Lhx9 proteins. A, Multiple protein sequence alignment showing that Lhx2 and Lhx9 are highly conserved across different mammalian and sauropsid species. Three functional domains are colored and framed. Seven different sites within the effector domains (LIM1 and LIM2) between the two proteins are highlighted by red triangles. B, Disorder prediction for chicken Lhx2 and Lhx9.

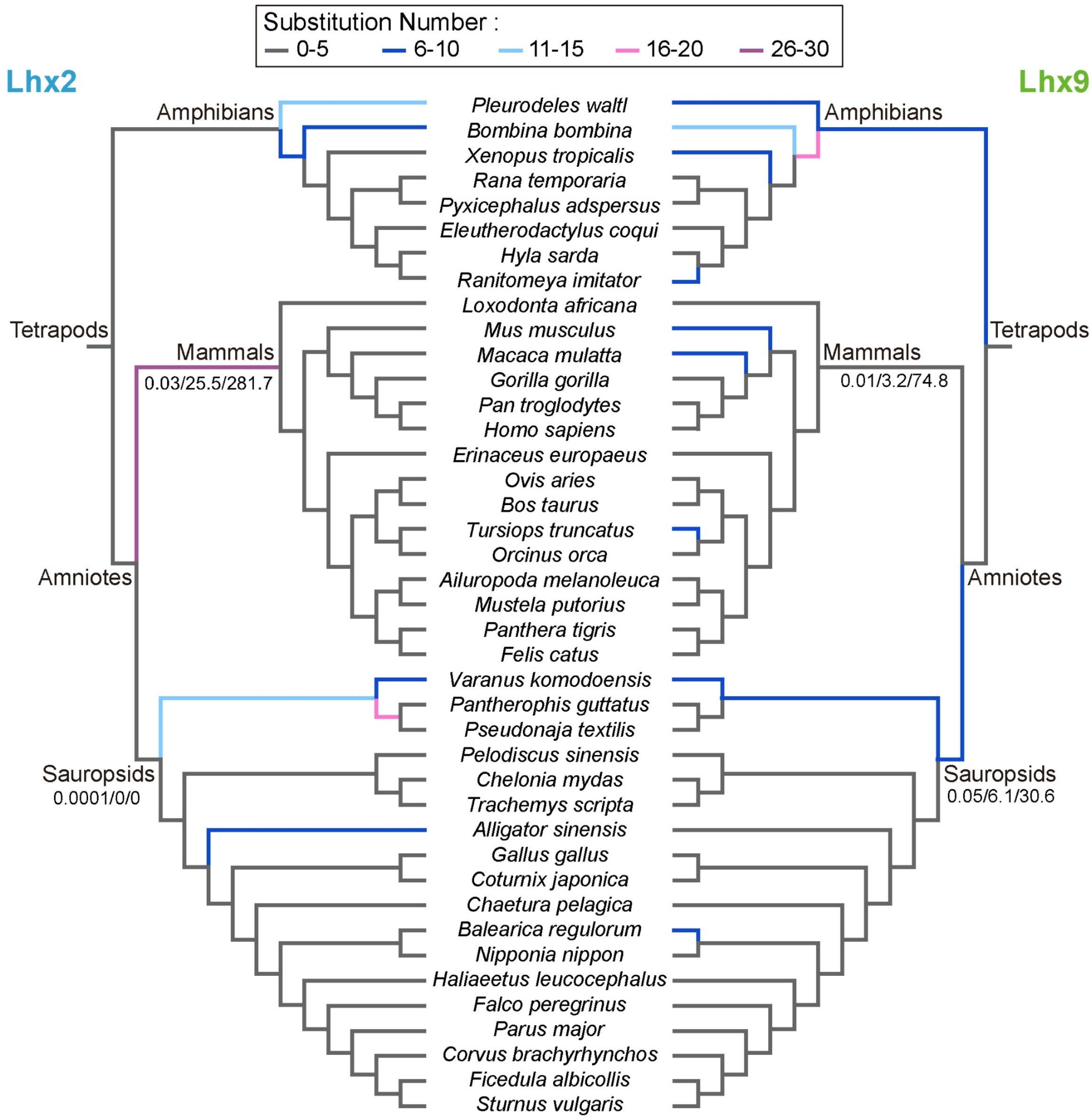

Fig. S9. Branch-wise amino acid substitution counts of Lhx2 and Lhx9 across tetrapods. On the mammalian and sauropsid branches, the three numbers indicate the ω values and the numbers of nonsynonymous and synonymous substitutions inferred from the free-ratio model.

Tables

Table S1. Molecular evolution of *Lhx2* and *Lhx9* in tetrapods.

Gene	Model (Focal clade)	Log-likelihood	Parameter	*P* - value
Lhx2	One-ratio	-7010.440542	ω_0 = 0.03	
	Two-ratio (Mammals)	-7009.700911	ω_0 = 0.03, ω_1 = 0.02	0.23
	Two-ratio (Sauropsids)	-7010.441207	ω_0 = 0.03, ω_1 = 2.35	0.97
	M2a_rel	-6950.578275	p_0 = 0.77, p_1 = 0.00, p_2 = 0.23<br>ω_0 = 0.00, ω_1 = 1.00, ω_2 = 0.10	
	Clade model C (Mammals)	-6940.874866	p_0 = 0.72, p_1 = 0.00, p_2 = 0.27<br>ω_0 = 0.00, ω_1 = 1.00,<br>ω_{2a} = 0.11, ω_{2b} = 0.03	< 0.001
	Clade model C (Sauropsids)	-6947.271512	p_0 = 0.76, p_1 = 0.00, p_2 = 0.24<br>ω_0 = 0.00, ω_1 = 1.00,<br>ω_{2a} = 0.08, ω_{2b} = 0.13	< 0.001
Lhx9	One-ratio	-7157.825417	ω_0 = 0.03	
	Two-ratio (Mammals)	-7156.475029	ω_0 = 0.03, ω_1 = 0.01	0.1
	Two-ratio (Sauropsids)	-7157.821243	ω_0 = 0.03, ω_1 = 0.03	0.92
	M2a_rel	-7039.660848	p_0 = 0.97, p_1 = 0.03, p_2 = 0.00<br>ω_0 = 0.02, ω_1 = 1.00, ω_2 = 29.86	
	Clade model C (Mammals)	-6968.29289	p_0 = 0.80, p_1 = 0.03, p_2 = 0.17<br>ω_0 = 0.00, ω_1 = 1.00,<br>ω_{2a} = 0.15, ω_{2b} = 0.05	< 0.001
	Clade model C (Sauropsids)	-6971.622396	p_0 = 0.83, p_1 = 0.02, p_2 = 0.15<br>ω_0 = 0.01, ω_1 = 1.00,<br>ω_{2a} = 0.12, ω_{2b} = 0.20	< 0.05

Table S2. Primers for amplifying WISH probes.

Gene	Forward (5' - 3')	Reverse (5' - 3')	Length (bp)
f*Lhx2*	CTGTCCAGAAAGGGAGACCGAG	CCAAGTTGCCAGGCACCG	513
c*Lhx2*	GTCCATCCAGCACTCCCACT	ACTGTAGCCATTACGAAGTAGTTTT	675
t*Lhx2*	TATTACAACGGCGTGGGGAC	AGATTTGTGAGAGTTGTCTGTGAAG	578
m*Lhx2*	GCACGCCTACAACCCTCACA	TATCCGAACAGACTTCCACCTTAA	695
r*Lhx2*	GCGCAACCTCTTACGG	CAAGTAATTCCACAGTCCG	454
b*Lhx2*	CCTGTTTCAGCAAGGATGG	CGTGTTTTCCTGCCGTAAG	605
f*Lhx9*	GAGGTGTGCCCGTTGC	CAAAACTCTCTTTGTTAATCCAGT	541
c*Lhx9*	CAGATATTACCTGCTGGCTG	GTTCGTGGCTGTCCAAGTTA	914
t*Lhx9*	TGCTGGTTGTGGAGGGAAG	GGCTGCTGGTCTCTGTCC	567
m*Lhx9*	CGACCATTTCGGGATGAAG	CTACAGTGACAGTGGGATTGG	605
r*Lhx9*	CCATTTCGGCATGAAGG	CTACAGTGATAGTGGGATTGG	602
b*Lhx9*	CAAGGAGGATTACTACAGGTTC	CCAAGATTTGTTCTCCCTG	590
c*Gli3*	TGTACCAATTGATGCGCGACA	GCGTCTCGATGACGTAGAAGAGCT	656
c*Hoxd11*	GTTCAACGCATTGAAGCCTCC	TGCCGGTCAGTGAGGTTGAG	802
c*Alx4*	GCCCGAGTACAGGTCTGGTTC	GTGCTCCTTCGCCTTCATCC	438
c*Fgf8*	CCCTGCTCCTCGCTCTTC	GCTCGGTGGTCTGGTGGC	553
c*Gdf5*	GACTCTCTCAGATGCAGAAAGA	ATCCAGTCATCCCAGCCC	714

f, frog; c, chicken; t, turtle; m, mouse; r, rabbit; b, bat.

Table S3. Accession numbers (*No.*) of the sequences used in this study.

**Species name**	***Lhx2* accession *No.***	***Lhx9* accession *No.***
Ailuropoda melanoleuca	XM_002919998.4	XM_034666982.1
Alligator sinensis	XM_014526802.2	XM_006025512.3
Balearica regulorum	XM_075772363.1	XM_075759071.1
Bombina bombina	XM_053696189.1	XM_053693506.1
Bos taurus	NM_001191175.1	XM_024976206.2
Chaetura pelagica	XM_009998823.1	XM_009999898.1
Chelonia mydas	XM_007060957.3	XM_043520961.1
Corvus brachyrhynchos	XM_008642140.2	XM_008630209.2
Coturnix japonica	XM_015879387.2	XM_015869354.2
Eleutherodactylus coqui	XM_066579779.1	XM_066597187.1
Erinaceus europaeus	XM_060200158.1	XM_016184915.2
Falco peregrinus	XM_005231418.3	XM_055815804.1
Felis catus	XM_042964501.1	XM_007096100.2
Ficedula albicollis	XM_005055727.1	XM_005050517.2
Gallus gallus	XM_046901651.1	NM_205426.2
Gorilla gorilla	XM_004048582.5	XM_019037641.4
Haliaeetus leucocephalus	XM_010570715.1	XM_010563404.1
Homo sapiens	NM_004789.4	NM_020204.3
Hyla sarda	XM_056539367.1	XM_056523459.1
Loxodonta africana	XM_064290884.1	XM_010590187.3
Macaca mulatta	NM_001261792.1	XM_015120769.3
Mus musculus	NM_010710.5	NM_001042577.1
Mustela putorius	XM_004757347.2	XM_004756215.2
Nipponia nippon	XM_009477025.1	XM_009474419.1
Orcinus orca	XM_004269241.3	XM_004285829.3
Ovis aries	XM_042245720.2	XM_042229301.2
Pan troglodytes	XM_016961627.3	XM_009440355.5
Panthera tigris	XM_042964501.1	XM_007096100.2
Pantherophis guttatus	XM_060682049.1	XM_034437459.2
Parus major	XM_015644574.1	XM_015636108.3
Pelodiscus sinensis	XM_075905658.1	XM_006124648.4
Pleurodeles waltl	XM_069241714.1	XM_069232118.1
Pseudonaja textilis	XM_026712460.1	XM_026700135.1
Pyxicephalus adspersus	XM_072430470.1	XM_072420050.1
Rana temporaria	XM_040324856.1	XM_040359876.1
Ranitomeya imitator	XM_069748458.1	XM_069737355.1
Sturnus vulgaris	XM_014880007.1	XM_014883354.1
Trachemys scripta	XM_034751974.1	XM_034779602.1
Tursiops truncatus	XM_033858575.1	XM_019920329.2
Varanus komodoensis	XM_044440011.1	XM_044432492.1
Xenopus tropicalis	XM_002938749.5	XM_004913744.4
Ailuropoda melanoleuca	XM_002919998.4	XM_034666982.1
Alligator sinensis	XM_014526802.2	XM_006025512.3
Balearica regulorum	XM_075772363.1	XM_075759071.1
Bombina bombina	XM_053696189.1	XM_053693506.1

Table S4. Primers for amplifying chicken full-length CDS.

Gene	Forward (5'- 3')	Reverse (5'- 3')	Length (bp)
Lhx2	ATGCTTTTCCACAGCCTGTCC	GACAACTCTCACAAATCTTTTCTGA	1203
Lhx9	ATGGAAATAGTGGGGTGCAGAG	TTAGAAAAGGTTCGTTAAGGTAGTTTG	1194

Table S5. Primers for RT-qPCR.

Gene	Forward (5' - 3')	Reverse (5' - 3')	Length (bp)
Lhx2	ATGAGGGCCAGGGATTT	GCGGCAATACACCAGATT	114
Lhx9	CTGGGTGTGGAGGGAAG	GGCAAAGCAGGTGAGTTC	125
Runx2	TGGGAACGACGAGAACTA	GGTATGTAGCGACTTGGG	167
Chrdl1	AGACAAGAAGTACCGTGTAGGA	GGGAAGGACAGTGTAGGCT	143
Gata6	GAGCTGCTGGAAGACCTCTC	GCTCAGGCCGTTCATTTTGG	138
Sulf1	CATCCTTCATCAATGCCTTCG	CCAGGAGGGAGAAGAGCAGTT	128
Notch1	AACGCTGTGGATGATCTGGG	AGCGGGGTCTCCTCCTTATT	131
Notch2	CACACCCGAGTGCTTGTTTG	ATTACAGCCCTGGTCACAGC	115
Hes4	GAAGTCCTCCAAACCCATCA	GCACTCATTGAAACCAGCC	247
Msx1	CTGCATTCGGCATCTCCTTCC	CGGCTCGGCCCTATGTAA	149
Hes1	GGACGCGCTGAAGAAGGATA	CTTCGCAGGTGGAGAGGAAC	203
Gli3	AGAAGCAGCGAGGAGACA	CACGCCTTGAGGTAGTGC	141
Alx4	CGGACCCACTTCTCCACT	AACACAAGCAGGCACAGG	123
Fgf8	GAGCAGAGCCTGGTGACA	GATGTAGAACCCCGTGGC	213
Hoxd11	GAAGAAAGTGACAACGGCGA	GGTCAGTGAGGTTGAGCATTCT	215

Dataset S1 (separate file). DEGs in *Lhx2*- and *Lhx9*-overexpressing limbs, and their overlapping genes.

Dataset S2 (separate file). GO-BP terms of DEGs after *Lhx2* and *Lhx9* overexpression.

SI References

1. S. Li *et al.*, *Zic3* represses anterior digit development in tetrapods. *Zool. Res.* **46**, 684–694 (2025).
2. M. Salinas-Saavedra *et al.*, New developmental evidence supports a homeotic frameshift of digit identity in the evolution of the bird wing. *Front. Zool.* **11**, 33 (2014).
3. A. Boczek-Funcke, J. P. Kuhtz-Buschbeck, J. Raethjen, B. Paschmeyer, M. Illert, Shaping of the cat paw for food taking and object manipulation: an X-ray analysis. *Eur. J. Neurosci.* **10**, 3885–3897 (1998).
4. A. Zuniga, Next generation limb development and evolution: old questions, new perspectives. *Development* **142**, 3810–3820 (2015).
5. Z. Wang, N. Han, P. A. Racey, B. Ru, G. He, A comparative study of prenatal development in *Miniopterus schreibersii fuliginosus*, *Hipposideros armiger* and *H. pratti*. *BMC Dev. Biol.* **10**, 10 (2010).
6. T. J. Zollinger, K. A. Backues, A. G. Burgos-Rodriguez, Correction of angular limb deformity in two subspecies of flamingo (*Phoenicopterus ruber*) utilizing a transphyseal bridging technique. *J. Zoo Wildl. Med.* **36**, 689–697 (2005).
7. R. H. D. Lambeck *et al.*, Biometrics and migration of oystercatchers (*Haematopus ostralegus*) from the White Sea region (NW Russia). *Ringing & Migration* **16**, 140–158 (1995).
8. M. A. G. de Bakker *et al.*, Selection on phalanx development in the evolution of the bird wing. *Mol. Biol. Evol.* **38**, 4222–4237 (2021).
9. J. O'Connor *et al.*, Chicago Archaeopteryx informs on the early evolution of the avian bauplan. *Nature* **641**, 1201–1207 (2025).
10. F. Lima *et al.*, Skeletogenesis of the pectoral girdle and forelimbs in embryos of Caiman yacare (Daudin, 1802)(Crocodylia, Alligatoridae). *J. Morph. Sci.* **28**, 4–10 (2011).
11. F. J. Tulenko, C. A. Sheil, Formation of the chondrocranium of *Trachemys scripta* (Reptilia: Testudines: Emydidae) and a comparison with other described turtle taxa. *J. Morphol.* **268**, 127–151 (2007).
12. M. R. Sánchez-Villagra, C. Mitgutsch, H. Nagashima, S. Kuratani, Autopodial development in the sea turtles *Chelonia mydas* and *Caretta caretta*. *Zoolog. Sci.* **24**, 257–263 (2007).
13. M. A. G. de Bakker *et al.*, Changes in evolutionary developmental control points in the amniote limb may explain hyperphalangy. *Mol. Biol. Evol.* **42**, msaf113 (2025).
14. S. Regnault, J. R. Hutchinson, M. E. Jones, Sesamoid bones in tuatara (*Sphenodon punctatus*) investigated with X‐ray microtomography, and implications for sesamoid evolution in Lepidosauria. *J. Morphol.* **278**, 62–72 (2017).
15. J. C. Ríos‐Orjuela, J. S. Camacho‐Bastidas, A. Jerez, Appendicular morphology and locomotor performance of two morphotypes of continental anoles: Anolis heterodermus and Anolis tolimensis. *J. Anat.* **236**, 252–273 (2020).
16. D. C. Blackburn, R. M. Keeffe, M. C. Vallejo-Pareja, J. Vélez-Juarbe, The earliest record of Caribbean frogs: a fossil coquí from Puerto Rico. *Biol. Lett.* **16**, 20190947 (2020).
17. J. Gao, X. Li, Y. Zhang, H. Wang, Endochondral ossification in hindlimbs during bufo gargarizans metamorphosis: A model of studying skeletal development in vertebrates. *Dev. Dyn.* **247**, 1121–1134 (2018).
18. Z. Shan *et al.*, Embryonic and skeletal development of the albino African clawed frog (*Xenopus laevis*). *J. Anat.* **242**, 1051–1066 (2023).
19. R. R. Kerney, J. Hanken, D. C. Blackburn, Early limb patterning in the direct-developing salamander *Plethodon cinereus* revealed by *sox9* and *col2a1*. *Evol. Dev.* **20**, 100–107 (2018).
20. N. B. Fröbisch, C. Bickelmann, F. Witzmann, Early evolution of limb regeneration in tetrapods: evidence from a 300-million-year-old amphibian. *Proc. Biol. Sci.* **281**, 20141550 (2014).
21. J. S. Anderson, R. R. Reisz, D. Scott, N. B. Fröbisch, S. S. Sumida, A stem batrachian from the Early Permian of Texas and the origin of frogs and salamanders. *Nature* **453**, 515–518 (2008).
22. F. J. Tulenko *et al.*, Fin-fold development in paddlefish and catshark and implications for

the evolution of the autopod. *Proc. Biol. Sci.* **284**, 20162780 (2017).
23. H. Hamada *et al.*, Pattern of fin rays along the antero-posterior axis based on their connection to distal radials. *Zoological lett.* **5**, 30 (2019).
